# Photosynthetic Adjustments Maintain Lettuce Growth Under Dynamically Changing Lighting in Controlled Indoor Farming Setups

**DOI:** 10.1111/ppl.70405

**Published:** 2025-07-12

**Authors:** Arttu Mäkinen, Hirofumi Ishihara, Sylvain Poque, Nina Sipari, Kristiina Himanen, Ilona Varjus, Juho Heininen, Matti Pastell, Paula Elomaa, Alexey Shapiguzov, Titta Kotilainen, Saijaliisa Kangasjärvi

**Affiliations:** ^1^ Department of Agricultural Sciences, Faculty of Agriculture and Forestry University of Helsinki Helsinki Finland; ^2^ Organismal and Evolutionary Biology Research Programme, Faculty of Biological and Environmental Sciences University of Helsinki Helsinki Finland; ^3^ National Plant Phenotyping Infrastructure, Helsinki Institute of Life Science, Biocenter Finland University of Helsinki Helsinki Finland; ^4^ Helsinki Metabolomics Center, Faculty of Medicine University of Helsinki Helsinki Finland; ^5^ Viikki Plant Science Centre University of Helsinki Helsinki Finland; ^6^ Division of Pharmaceutical Biosciences, Faculty of Pharmacy University of Helsinki Helsinki Finland; ^7^ Natural Resources Institute Finland Helsinki Finland; ^8^ Natural Resources Institute Finland Turku Finland

**Keywords:** controlled environment agriculture, LED‐lighting, lettuce, light acclimation, photosynthesis

## Abstract

Studies have uncovered delicate mechanisms that enable plant acclimation to fluctuating light. Translating the knowledge to controlled environment agriculture could advance the development of cost‐effective dynamic lighting strategies where the light intensity is purposely alternated, mirroring the spot electricity price, but its effects on vegetable crops remain poorly understood. Here, we recorded photosynthetic parameters, metabolic responses, and growth of lettuce ( *Lactuca sativa* L.) cv. “Katusa” under dynamic lighting. The light intensity was altered at different times of the photoperiod with uniform daily light integral. Three different setups, including a plant phenotyping facility, a small‐scale vertical farm testbed and a larger‐scale vertical farm, were utilized to address the physiological responses and scalability of lighting strategies. The lettuces readily adjusted their photosynthetic light reactions and carbon metabolism according to the changing light intensities. However, the overall metabolic composition of lettuce leaves did not respond to dynamic lighting. Upon simulation of commercial production in the larger‐scale vertical farm, constant and dynamic lighting regimes yielded lettuce heads with equal saleable sizes of 87–89 g, even under artificial “Split‐Night” regimes where the photoperiod was interrupted by two periods of darkness. These findings suggest that dynamic lighting strategies enable cost‐effective lighting via optimization of electricity use in indoor cultivation.

## 1 Introduction

Light is an important environmental factor that affects the productivity and nutritional value of crops. In natural conditions, wind‐induced canopy movements, cloudiness, and climatic and seasonal alterations affect the light environment at different time scales (Kaiser et al. 2019; Slattery et al. 2018; Kotilainen et al. 2020). Plants respond to changes in the direction, duration, intensity, and spectral quality of light, which in natural conditions alternate seasonally and within a 24‐h diurnal cycle (Brelsford et al. 2019; Sellaro et al. 2010), and are affected by neighboring plants (Ballaré et al. 1987; Casal 2013). Studies have uncovered fast photosynthetic rearrangements and more durable light‐induced metabolic and developmental adjustments, which may affect plant productivity (Morales and Kaiser 2020; Hotta 2021; Dantas et al. 2023; Poorter et al. 2019; Slattery et al. 2018; Kaiser et al. 2019). More recently, the application of changing light conditions has been recognized as a way to achieve cost‐effective lighting in controlled environment agriculture (CEA) (Kaiser et al. 2024; Bechtold et al. 2025; Lazzarin et al. 2024). In dynamic lighting strategies, the intensity of artificial lighting is purposely alternated during indoor cultivation.

Dynamic adjustment of lighting can be achieved with light‐emitting diode (LED)‐luminaires fitted with adjustable light intensity output capabilities that enable dimming. An intrinsic property of LEDs is that their light intensity output is dependent on the electrical current. Therefore, by regulating the supplied electrical current, their intensity and electricity consumption can be adjusted almost instantaneously. When paired with modern control and automation systems, dimmable LED luminaires allow adjustment of the light environment in indoor cultivation. The ability to dynamically adjust lighting is becoming increasingly important, since lighting and heating make up a significant part of the production costs in CEA, especially in wintertime at northern latitudes. European power markets have changed in recent years with record‐breaking levels and fluctuations in prices (Cevik and Ninomiya 2023). The Nord Pool power exchange (https://www.nordpoolgroup.com/) publishes hourly spot electricity prices daily, 24 h in advance, which would allow optimization of light intensity in CEA according to the electricity price. However, how varying light cycles affect crop quality and productivity remains insufficiently understood.

In photobiological studies, analysis of chlorophyll fluorescence (ChlF) enables non‐invasive assessment of photosynthetic performance. In photosynthetic light reactions, electrons flow from Photosystem II (PSII), cytochrome b6f complex (Cytbf) and photosystem I (PSI) to NADP^+^, generating reducing power in the form of NADPH. The electron transfer reactions also generate a proton motive force across the thylakoid membrane, which drives the production of ATP by the ATP synthase. In photosynthetic carbon metabolism, a variety of enzymes consume NADPH and ATP for carbon fixation, starch biosynthesis, and other biosynthetic processes (Stirbet et al. 2020). In a changing light environment, transient perturbations in photosynthetic electron transfer reactions may enhance photoinhibition of PSII, commonly measured as the ChlF parameter Fv/Fm (Baker 2008; Murchie and Lawson 2013; Järvi et al. 2015; Li et al. 2018). At the same time, acidification of the thylakoid lumen activates dissipation of excess excitation energy as heat at the light‐harvesting complex of PSII (LHCII), which can be measured as increased qE, the major component of non‐photochemical quenching of chlorophyll fluorescence (NPQ) (Niyogi et al. 1998; Ruban 2016; Walter and Kromdijk 2022). On a cellular level, chloroplast movement can optimize light harvesting, although the physiological role of light‐induced chloroplast relocation has recently been debated (Wilson and Ruban 2020).

Photobiological studies on the model plant *Arabidopsis thaliana* have shown that photosynthetic activities are sensitive to the frequency, duration, and magnitude of light intensity changes (Vialet‐Chabrand et al. 2017; Von Bismarck et al. 2023). Matthews et al. (2018) found that photosynthetic gas exchange responded to the intensity and pattern of changing light intensities and that the responses varied at different times of the day. Notably, reduced accumulation of biomass was a common response to stressful changes in light intensities when compared to plants grown under constant light intensity (Morales and Kaiser 2020).

Among commercially cultivated leafy vegetables, lettuce ( *Lactuca sativa* L.) was reported to tolerate moderate light intensity changes every 15 min without negative effects on growth, whereas more drastic, ten‐fold changes in light intensity or alternating light and dark episodes impaired photosynthetic activity and growth (Bhuiyan and van Iersel 2021). While the underlying molecular mechanisms remain unresolved, crop performance under different frequencies, durations, intensities, and spectrum of dynamic lighting should be examined to avoid stress‐induced growth reduction and formation of off‐flavor in commercial produce (Kaiser et al. 2024).

Here we tested a hypothesis that it is possible to develop dynamic lighting regimes that are cost‐effective and support lettuce cultivation in commercial production. To this end, we analysed the physiological performance and growth of lettuce cv. “Katusa” in three different cultivation setups, including a small‐scale vertical farm testbed, a high‐throughput plant phenotyping facility, and a larger‐scale experimental vertical farming system, which allowed testing the scalability of dynamic lighting strategies. The light intensity was varied at different times of the photoperiod, with uniform daily light integral (DLI) across different lighting regimes within each cultivation set‐up. We found that lettuce responded to dynamic lighting by delicate adjustments in photosynthetic light reactions and carbon metabolism, while the overall metabolite profiles remained largely unaltered. Our findings suggest that the application of dynamic lighting offers cost‐effective cultivation solutions that support lettuce growth without impairing its nutritional quality in controlled indoor farming setups.

## 2 Materials and Methods

### 2.1 Plant Material, Growth Conditions, and Lighting at the Seedling Phase

Lettuce ( *L. sativa* L. cv. “Katusa”) seeds were obtained from Puutarhaliike Helle Oy, Lieto, Finland. To test the effects of dynamic lighting in different growth environments, the plants were grown in three different experimental set‐ups, including a small‐scale vertical farm testbed constructed in‐house, National Plant Phenotyping Infrastructure (NaPPI; https://www.helsinki.fi/en/infrastructures/national‐plant‐phenotyping, PlantScreen Compact System, Photon Systems Instruments, PSI), and a larger‐scale vertical farming system (VIS, Vacuum Insulation Solutions Oy.). The growth conditions at the seedling stage and experimental phase are detailed in Table S1. Data arising from these different experimental setups were analysed independently of each other.

In experiments performed in a small‐scale vertical farm testbed, pots with stratified seeds were distributed to irrigation trays that were divided into four light‐isolated cultivation shelf compartments. For 9 days of initial growth, the conditions were set as follows: light intensity of 155 μmol m^−2^ s^−1^ photosynthetic photon flux density (PPFD), photoperiod of 18/6 h light/dark (L18:D6), air temperature during the day 21°C, and night 19°C, and relative humidity (RH %) of 40%–60%. The positions of the pots were randomly rearranged multiple times during the experiments to alleviate possible border effects and environmental gradients.

In the phenotyping experiment, pots with stratified seeds were distributed to 24 imaging trays positioned in eight light‐isolated cultivation shelf compartments (CS 250/300_4_2.4, PSI). For 12 days of initial growth, the conditions were as follows: light intensity 155 μmol m^−2^ s^−1^ PPFD, photoperiod L18:D6, air temperature during the day 21°C, and night 19°C, and RH 60%–70%.

The larger‐scale vertical farming system that was set up to simulate commercial production was located at the Natural Resources Institute Finland research station in Piikkiö, Finland. The seeds were germinated in darkness in a climate‐controlled dark room for 1 day at 18°C and thereafter transferred to a seedling line in a standard greenhouse under automatically controlled climatic conditions (Itumic). For 2 weeks of initial growth, the conditions were set as follows: light intensity 350 μmol m^−2^ s^−1^ PPFD, photoperiod L18:D6, air temperature 18°C, relative humidity 60%–80%, and 800 ppm of CO_2_. After the initial growth stage, 264 plants per experiment (144 for imaging and 120 for final harvest measurements) were transferred into a hydroponic nutrient film technique‐cultivation (NFT) system located in the larger‐scale vertical system divided into three light‐isolated compartments.

### 2.2 Lighting Regimes and Spectral Composition During Plant Growth at the Experimental Phase

After initial growth at constant light intensity, the plants were shifted to different lighting regimes (Table S1). Adjustable (0%–100%) and independently programmable digital LED‐light controllers connected to dimmable LED‐lights were used to implement the varying light intensity regimes in all three experimental setups. In the plant phenotyping experiment, LED‐light controllers embedded in NaPPI cultivation shelves (LC, PSI) were used, whereas in the small‐scale vertical farm testbed and the larger‐scale system, a universal LED‐light control system (GrowFlux Universal Dimmer; GrowFlux Access Point, GrowFlux Ltd.) was used. Light intensity target values at canopy level were verified using a PAR‐light meter (LI‐250A Light Meter and Quantum Photometric Sensor, LI‐COR Inc.).

**FIGURE 1:**
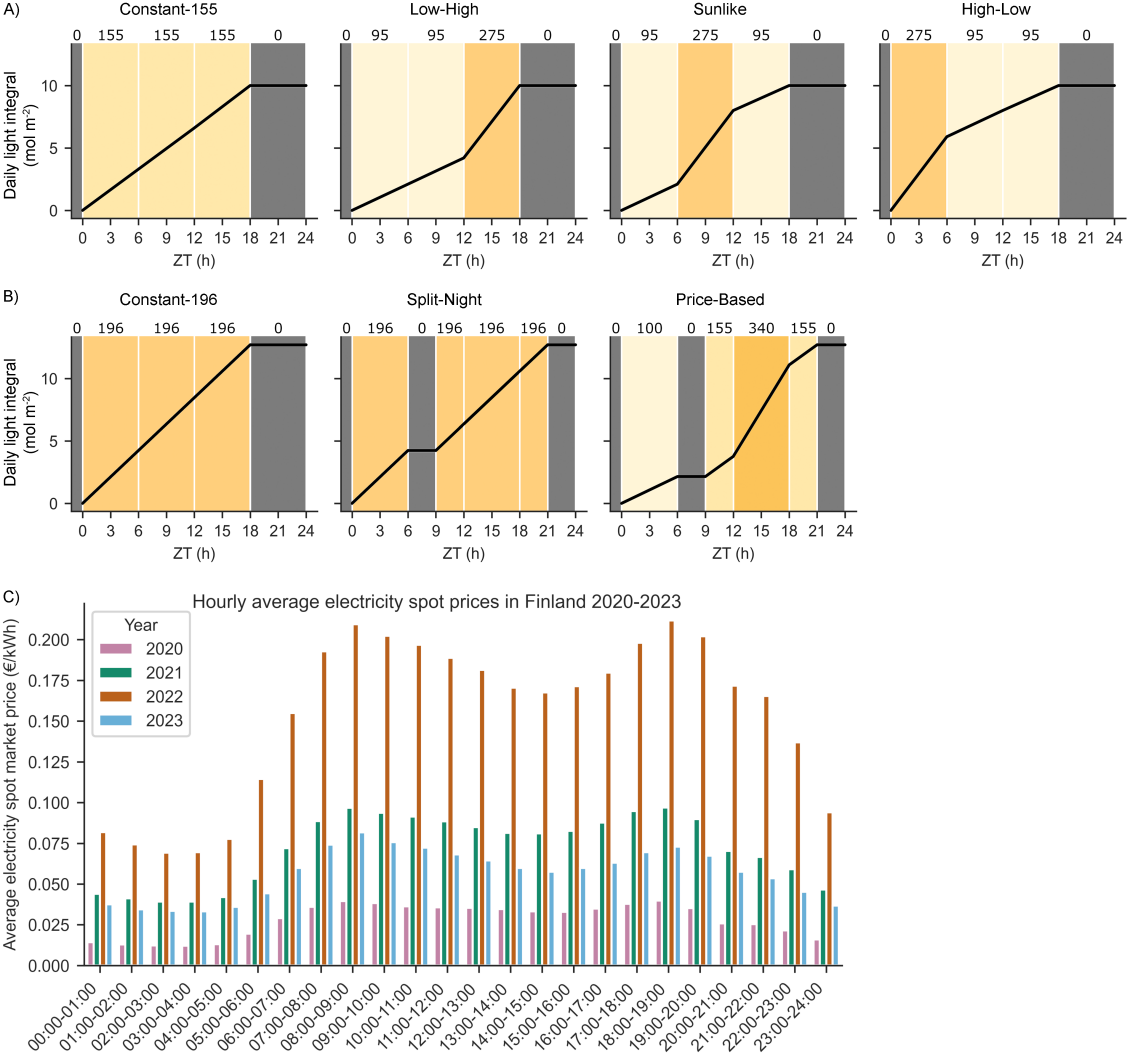
Lighting regimes used in experiments on lettuce cv. “Katusa”. Black lines indicate cumulative daily light integral (DLI) values, numbers on top of the panels and different shades of the background color indicate target light intensity values (PPFD). ZT (h) indicates time in hours elapsed since the beginning of the photoperiod. (A) In a small‐scale vertical farm testbed and plant phenotyping platform, the lighting regimes were termed Constant‐155, Low‐High, Sunlike and High‐Low, with an equal DLI of 10.0 mol m^−2^ PPFD, and the photoperiod was scheduled to start at 06:00. (B) In a larger‐scale vertical farming experiment, the lighting regimes were termed Constant‐196, Split‐Night and Price‐Based, with different light intensity levels but an equal DLI of 12.7 mol m^−2^ PPFD, and the photoperiod was scheduled to start at 11:00 to match the dark periods in Split‐Night and Price‐Based with hours of high electricity price. (C) Hourly changes in average electricity market spot price (€/kWh) during a 24‐h period in Finland between 2020 and 2023.

Lighting implemented in the small‐scale testbed and the plant phenotyping experiments consisted of four lighting regimes, termed “Constant‐155”, “High‐Low”, “Sunlike” and “Low‐High” (Figure 1A). All lighting regimes were set to start at 06:00 and consisted of a L18:D6 photoperiod with a cumulative DLI of 10 mol m^−2^ PPFD. In “Constant‐155”, an 18‐h photoperiod with light intensity of 155 μmol m^−2^ s^−1^ PPFD was used to emulate a commonly used lighting strategy in leafy vegetable production. In the other three lighting regimes, the 18‐h photoperiod was divided into three 6‐h periods; 0–6, 6–12, and 12–18 h ZT, in which ZT indicates the time elapsed since the onset of photoperiod (from the German word *zeitgeber* for “time‐giver”). During these periods the light intensity was set either to low light (95 μmol m^−2^ s^−1^ PPFD) or high light (275 μmol m^−2^ s^−1^ PPFD) at specific times of the photoperiod. In High‐Low lighting regime, high light was applied at 0–6 h ZT, in Sunlike at 6–12 h ZT, and in Low‐High at 12–18 h ZT of the photoperiod. During the last 6‐h period, at 18–24 h ZT, the light intensity was set to 0 μmol m^−2^ s^−1^ PPFD (darkness) in all regimes.

Lighting in the larger‐scale vertical farming experiment consisted of three lighting regimes, termed “Constant‐196”, “Split‐Night” and “Price‐Based” (Figure 1B). All lighting regimes were set to start at 11:00 and consisted of photoperiods with a cumulative DLI of 12.7 mol m^−2^ PPFD. Constant‐196 consisted of L18:D6 at 196 μmol m^−2^ s^−1^, Split‐Night was L6:D3:L12:D3 with lighting at 196 μmol m^−2^ s^−1^, and Price‐Based was L6:D3:L12:D3 with a more complex profile of 6 h of light at 100 μmol m^−2^ s^−1^, 3 h of darkness, 3 h of light at 150 μmol m^−2^ s^−1^, 6 h of light at 340 μmol m^−2^ s^−1^, 3 h of light at 150 μmol m^−2^ s^−1^, and 3 h of darkness. Since the photoperiods were programmed to start at 11:00, the two dark periods, at 6–9 h ZT and 21–24 h ZT in Split‐Night and Price‐Based regimes, coincided with the expensive hours at 08:00–11:00 a.m. and 17:00–20:00 p.m. (Figure 1B,C). The light intensities thereby followed a pattern that reflected changing electricity market prices during a 24‐h cycle (Figure 1C).

The three experimental setups differed with respect to the spectral composition of illumination. In the small‐scale vertical farm testbed, we used Valoya AP673L spectrum (BX120, Valoya Greenlux Lighting Solutions Oy.) (Figure S1). The plant phenotyping experiment was conducted under a PSI spectrum (CS 250/300_4_2.4, PSI, Drásov, Czech R.). In the larger‐scale experiment, we used the PhysioSpec Greenhouse spectrum (VYPR, Fluence Bioengineering Ltd.) for initial growth and Valoya Solray spectrum (BX120, Valoya Greenlux Lighting Solutions Oy.) (Figure S1). The spectral photon irradiance was measured with an array spectroradiometer (Maya2000 Pro Ocean Optics; D7‐H‐SMA cosine diffuser, Bentham Instruments Ltd.) (Figure S1). Measurements were recorded within the wavelength range 315–800 nm and processed in R (R Core Team 2017), using the photobiology packages developed for spectral analysis (Aphalo 2015).

### 2.3 Calculation of Potential Electricity Cost Savings

**FIGURE 2:**
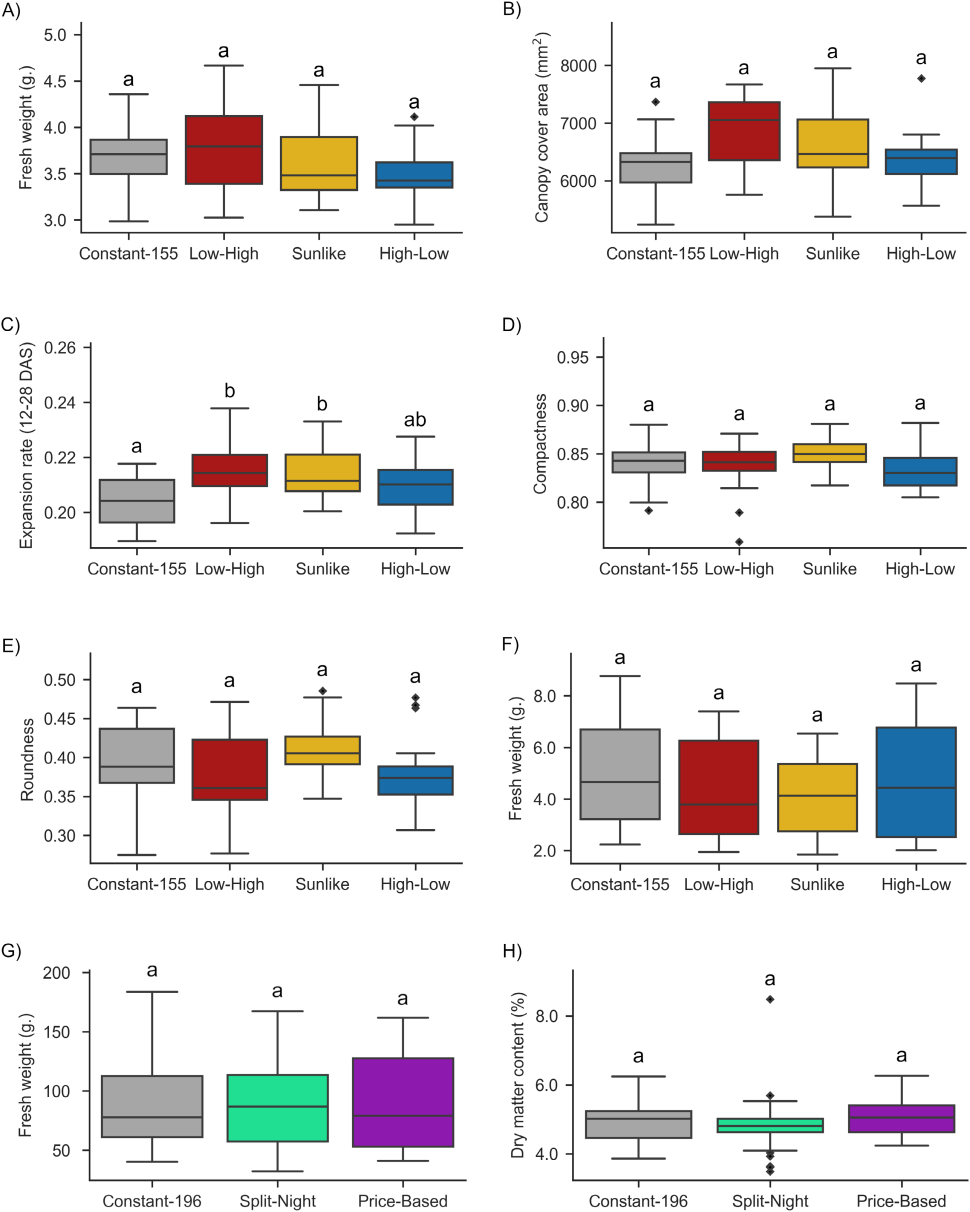
Lettuce cv. “Katusa” growth parameters measured in the plant phenotyping facility (*n* = 2) (A–E), small‐scale vertical farming testbed (*n* = 3) (F), and larger‐scale vertical farm (*n* = 3) (G–H). Fresh weights and dry weights were measured manually using a scale, and canopy cover area, canopy cover area expansion rate, compactness and roundness were calculated from top‐view RGB‐images. The data were analysed by fitting a linear mixed effects model, with random effects for the block/shelf/experiment grouping factors. The letters indicate significant differences between lighting treatments at *p* < 0.10 for pairwise comparisons, which were performed only when overall significance of the treatments yielded *p* < 0.05. *p*‐values from multiple contrasts were adjusted using Holm's procedure. *p* = 0.05 was used as the limit for significance of treatment effect, while for pairwise comparisons *p* = 0.10 was used.

The potential electricity cost savings of different lighting regimes were calculated using the following method (as described in detail in Data S1): (1) The hourly dynamics of average electricity price fluctuations within a 24‐h period (in €/kWh) for different years were calculated using historical electricity spot price data between 2020 and 2023 in Finland (https://www.nordpoolgroup.com/en/elspot‐price‐curves/). The prices were at their lowest during late night hours (23:00–05:00) and highest during morning (08:00–11:00) or afternoon/early evening (17:00–20:00) (Figure 1C); (2) The average price fluctuation patterns were used to identify, rank, and target the hours of the day which yield the highest reductions in electricity costs; (3) The hourly target light intensity values of the lighting regimes between experiments (Figure 1A,B) were scaled to an equal DLI and then used in conjunction with the corresponding measured electricity consumption data of the LED‐lights (in kWh) to find the best fit with the lowest electricity prices; (4) The total hourly electricity costs of different lighting regimes for a 24‐h period were calculated throughout the photoperiod; (5) Finally, the total daily electricity costs for illuminating one square meter area (€/m^2^) of the constant and dynamic lighting regimes were calculated to allow percentage‐wise comparison of potential electricity cost savings. As a baseline for all comparisons, a constant lighting regime with a photoperiod set between 05:00–23:00 (L18:6D) was used.

### 2.4 Manual Analysis of Growth

In all experimental setups, above‐ground plant fresh weights (FW) were measured immediately after harvesting using a digital scale. In the larger‐scale vertical farming experiment, the samples were oven‐dried at +60°C for 7–14 days, after which their dry weights (DW) were measured.

### 2.5 Image‐Based Analysis of Growth, Color and Chlorophyll Fluorescence

In the plant phenotyping experiment in NaPPI, visible light RGB imaging was used to record individual plant growth, roundness, compactness, and color under the different lighting regimes. Plants were transferred to the imaging station, and top‐view images were captured at 8, 12, 16, 20, 24, and 28 days after sowing (DAS) to record changes in canopy cover area over time. PlantScreen Data Analyzer v3.2.4.5 (PSI) was employed to post‐process the RGB images. The plant surface area was segmented using pixel color thresholding for RGB images. Roundness, compactness, and physiological parameters were extracted automatically by PlantScreen Data Analyzer v3.2.4.5 (PSI). Plant canopy area expansion rates (mm^2^ · day) from 12 DAS to 28 DAS were determined using the following equation:

$$\text{Canopy area expansion rate}=\frac{\bar{\ln\left(A_{2}\right)}-\bar{\ln\left(A_{1}\right)}}{t_{2}-t_{1}}$$

where $\bar{\ln{\left(A\right)}_{t}}$ is the averaged natural logarithm‐transformed canopy cover area at time t; $A_{2}$ and $A_{1}$ are plant canopy cover areas at $t_{2}$ and $t_{1}$, respectively; and $t_{2}$ and $t_{1}$ are harvest days 28 and 12 DAS.

Canopy Greenness was computed from RGB images using Python version 3.10.7 and OpenCV (CV2, https://opencv.org/). Pixel values for Red (R), Green (G), and Blue (B) channels were averaged to obtain the average Red (avgR), Green (avgG), and Blue (avgB) values. The Greenness was then determined using the formula by Signorelli et al. (2023):

$$\text{GREENNESS}=\frac{2\times \mathrm{avg}G-\mathrm{avg}R-\mathrm{avg}B}{2\times \mathrm{avg}R+\mathrm{avg}G+\mathrm{avg}B}$$

ChlF and canopy leaf greenness were analysed between 27 and 28 DAS, 30 min before the light intensity changes took place at 0, 6, 12, and 18 h ZT. ChlF measurements were performed using a FluorCam FC‐800MF pulse amplitude modulated (PAM) chlorophyll fluorometer (PSI.). ChlF parameters were determined after a 20‐min dark period using a quenching protocol developed with FluorCam 7.0 software (PSI). The quenching protocol consisted of three phases: initial dark phase (21.7 s), actinic light phase (70 s), and final dark phase (100 s). In the initial dark phase, the first measurement was taken at 5.06 s by applying an 800 ms saturating pulse at 1085 μmol m^−2^ s^−1^ to determine the maximum fluorescence Fm. NPQ was calculated as (Fm − Fm′)/Fm′ where Fm′ is the maximum fluorescence yield in light‐acclimated conditions (Horton and Ruban 1992). In the actinic light phase with 155 μmol m^−2^ s^−1^, five saturating pulses at 31.7, 41.7, 51.7, 71.7, and 91.7 s were applied to measure fluorescence levels at L1, L2, L3, L4 and Lss, respectively. In the final dark phase, three saturating pulses were applied at 121.7, 181.7, and 191.7 s to measure fluorescence levels at D1, D2, and D3, respectively. Camera shutter speed and sensitivity were set to 33 μs and 5%, respectively.

In the larger‐scale vertical farming experiment, ChlF imaging was performed on the last day of the experiment (35 DAS) at a two‐hour interval between 0 and 24 h ZT using the PlantScreen SC Mobile System (PSI). The ChlF protocol was executed after a 30‐min dark acclimation and included a 9‐min high light period (800 μmol m^−2^ s^−1^) followed by a 27‐min dark relaxation and then a light ramp consisting of 3‐min steps of increasing light intensity (100, 200, 300, 600, 800, 1000 and 1200 μmol m^−2^ s^−1^). Saturating light pulses with a duration of 800 ms and intensity ca. 3000 μmol m^−2^ s^−1^ at the plant level were triggered every 3 min throughout the protocol to determine quantum yields of PSII and NPQ. Maximum quantum efficiency of PSII photochemistry (Fv/Fm) was calculated as Fv/Fm = (Fm − Fo)/Fm, where Fo and Fm are maximum and minimum fluorescence yields, respectively (Butler 1978). Values for NPQ were calculated as (Fm − Fm′)/Fm′ (Horton and Ruban 1992) at 9 min into the dark relaxation period and were termed Partially relaxed NPQ. The ChlF data was post‐processed using Fluorcam10 software (PSI). Plant segmentation using pixel color thresholding followed the same principle as for the plant phenotyping experiment, while data extraction was conducted with a custom processing pipeline.

### 2.6 Analysis of Metabolites

For the analysis of soluble carbohydrates and starch in the small‐scale vertical farming experiment, samples were harvested at the end of the experiment (23 DAS) at a 6‐h timepoint interval before the light intensity changes took place at 0, 6, 12, and 18 h ZT. From each lighting regime, 3 individual plants per timepoint were collected. In the larger‐scale vertical farming experiment, samples were harvested at the end of the experiment (35 DAS) at a 2‐h timepoint interval between 0 and 24 h ZT. From each lighting regime, three replicate sets of 24 leaf disks (diameter 8 mm) from a pool of 4 plants (6 disks per plant) per timepoint were collected. Immediately after harvesting, the samples were placed in vials, flash‐frozen in liquid nitrogen, and stored at −80°C. Frozen samples were ground to a fine powder with a ball‐mill (Retsch MM 400 Mixer Mill, Retsch GmbH). For the analysis of glucose, fructose, sucrose, and starch concentrations, metabolites were extracted twice with 80% ethanol and once with 50% ethanol from 20 mg of frozen tissue powder and analyzed as described in Cross et al. (2006).

For large‐scale metabolite profiling, metabolites were extracted from approximately 50 mg (FW) of plant material per sample as described in Sipari et al. (2022) (for details, see Data S2). Feature extraction from raw LC–MS data was conducted using MZmine (Version 4.0.3) in batch mode, and the resulting features were further processed with Python (Version 3.11.3). The features were filtered based on retention time (0.4–4 min) and a minimum feature intensity of 10 across all samples. Additionally, features were required to have a relative standard deviation (RSD) of less than 150% across QC samples and to be present in at least two‐thirds of the samples within a sample class. Unsupervised dimension reduction was done using principal component analysis (PCA). For PCA, QC samples were excluded from the dataset, and the missing values were imputed using the lowest 10% of feature‐wise intensity. The dataset was then log‐transformed and Z‐score normalized. The normalized data was projected onto the first two principal components (PC1 and PC2).

### 2.7 Statistical Analyses

All three experimental setups were based on randomized complete block design (RCBD), with different lighting regimes regarded as experimental units. Time‐independent experiment repetitions, growth chamber shelf units, and compartments used in the experiments were regarded as block grouping factors. Individual plant replicates were regarded as subsamples or observational units.

The small‐scale vertical farming experiment was repeated three times. Plants in each lighting regime were grown on separate shelves, and each time‐independent repetition with 12–24 subsamples was regarded as a block (*n* = 3). The plant phenotyping experiment in NaPPI was conducted once. Plants in each lighting regime were grown on two separate shelves, and each shelf unit with 10 subsamples was regarded as a block (*n* = 2). The larger‐scale vertical farming experiment was repeated three times. Plants in each lighting regime were grown in three separate compartments on a rotating basis between repetitions, and each time‐independent repetition with 35–40 subsamples was regarded as a block (*n* = 3).

Data for leaf FW and DW, canopy cover area, roundness and compactness, soluble carbohydrates and chlorophyll fluorescence parameters were analysed by fitting a linear mixed effects model, with random effects for the block/shelf/experiment grouping factors. Data obtained from experiments conducted in different experimental setups were analysed independently. Analysis was performed with R software (R Core Team 2024), using package NLME (Pinheiro and Bates 2000; Pinheiro et al. 2023). In cases when variances were not homogeneous, a power of variance function covariate was included in the model. If the test of overall significance of the treatments yielded *p* < 0.05, comparisons between individual pairs of treatments were done with package GMODELS (Warnes et al. 2024) to fit these contrasts. *p*‐values from multiple contrasts were adjusted using Holm's procedure. *p* = 0.05 was used as the limit for significance of treatment effect, while for pairwise comparisons *p* = 0.10 was used. In cases where the linear mixed effects model was not applicable, differences between lighting regimes at individual timepoints were analysed separately using ANOVA and Tukey HSD post hoc test with Bonferroni correction for pairwise comparisons. This included data for chlorophyll fluorescence parameters from the plant phenotyping experiment.

## 3 Results

### 3.1 Electricity Spot Market Price Fluctuations Can Be Exploited to Decrease the Electricity Costs of Artificial Lighting

Analysis of electricity spot market price data between 2020 and 2023 revealed large annual and daily variations in electricity prices in Finland (Figure 1C). In all years, the prices were at their lowest during late night hours (23:00–05:00) and highest during morning (08:00–11:00) or afternoon/early evening (17:00–20:00) (Figure 1C). This repeating pattern allowed the identification and ranking of the most suitable hours in terms of potential electricity cost savings, and a comparison analysis between constant and dynamic lighting regimes with equal DLI. Our analysis showed that the dynamic lighting regimes could have resulted in consistently lower electricity costs than constant lighting regimes during 2020–2023 (Table 1). Dynamic lighting regimes yielded on average 29.8% reductions in electricity costs each year compared to a baseline constant lighting regime (Table 1). The highest cost savings in dynamic lighting regimes were achieved by Price‐Based in 2020 (38.3%), and the lowest by Split‐Night in 2023 (19.9%). Low‐High, Sunlike, and High‐Low resulted in intermediate cost savings each year but repeatedly surpassed the constant lighting regime baseline cost savings.

**TABLE 1:** Total electricity costs (€/m^2^) and percentage‐wise comparison of the potential cost savings between constant and dynamic lighting regimes with an electricity spot market price‐based plan during 2020–2023.

Lighting regime	Total electricity cost (€/m^2^)				Percentage‐wise cost comparison			
	2020	2021	2022	2023	2020	2021	2022	2023
Constant 05‐23	0.040 €	0.099 €	0.215 €	0.077 €	Baseline	Baseline	Baseline	Baseline
Constant 11‐05	0.032 €	0.083 €	0.175 €	0.064 €	20.0%	16.4%	18.8%	17.1%
Constant 17‐11	0.031 €	0.082 €	0.175 €	0.066 €	21.3%	17.2%	18.4%	14.0%
Constant 23‐17	0.032 €	0.084 €	0.174 €	0.066 €	17.8%	15.2%	19.2%	13.5%
Low‐High	0.026 €	0.070 €	0.143 €	0.055 €	35.1%	29.0%	33.4%	27.8%
Sunlike	0.025 €	0.070 €	0.144 €	0.057 €	36.0%	29.4%	33.1%	25.9%
High‐Low	0.026 €	0.071 €	0.143 €	0.057 €	33.8%	28.2%	33.6%	25.5%
Split‐Night	0.030 €	0.078 €	0.165 €	0.061 €	24.8%	21.0%	23.4%	19.9%
Price‐Based	0.024 €	0.067 €	0.138 €	0.054 €	38.3%	32.0%	35.9%	29.3%

*Note:* Numbers 05–23, 11–05, 17–11 and 23–17 in the Constant lighting regimes indicate photoperiod scheduling times in hours during a 24‐h period. Lighting regime Constant 05–23 was used as a baseline for all comparisons.

We also examined the effect of simply shifting the photoperiod of a constant lighting regime toward lower demand hours and calculated the potential electricity costs in different photoperiod starting time scenarios. Compared to a baseline constant lighting regime, shifting the photoperiod by 6–18 h decreased the electricity costs by 14.9%–19.7% on average. The best starting time for constant lighting regimes varied by year, with the highest cost savings achieved by a photoperiod set between 17:00–11:00 in 2020 and 2021 (21.3 and 17.2%, respectively), 23:00–17:00 in 2022 (18.4%), and 11:00–05:00 in 2023 (17.1%).

### 3.2 Dynamic Lighting Regimes Support Lettuce cv. “Katusa” Growth in Different Indoor Cultivation Setups

Testing lettuce cv. “Katusa” growth in the plant phenotyping platform did not reveal significant differences in plant FW at the end of the experiment 29 DAS (Figure 2A). Accordingly, assessment of seedling growth by RGB imaging of canopy cover area at the end of the experiment at 29 DAS did not reveal statistically significant differences between lighting regimes (Figure 2B). Canopy cover area expansion rates (mm^2^ day^−1^) between 12 DAS and 28 DAS were, however, observed to be higher in Low‐High, Sunlike and High‐Low than in Constant‐155 (Figure 2C). Plant morphological characteristics measured as canopy roundness and compactness were not affected by the lighting regimes (Figure 2D,E). Also in the small‐scale vertical farm, the lettuces displayed similar FW at 23 DAS in all the lighting conditions studied (Figure 2F).

Next, we followed lettuce growth under Constant‐196 and the cost‐effective Split‐Night and Price‐Based regimes in a larger‐scale vertical farming system (Table 1), which enabled testing under conditions that could be scaled up to commercial production (Table S1). The plants were grown for up to 35 days to reach a larger head size, which represents saleable sizes in commercial lettuce cultivation. Neither Split‐Night nor Price‐Based affected lettuce growth when compared to growth under constant light, as indicated by comparable FW at 87–89 g and dry matter contents of 4.8%–5.0% observed at harvest (Figure 2G,H). Flowering was not induced in any of the conditions studied. These findings suggested bipartite Split‐Night regimes with dynamic light intensities as a promising tool to optimize resource use efficiency in lettuce production.

### 3.3 Quantum Efficiency of Photosynthesis and NPQ Respond Dynamically to Alternative Lighting Regimes

**FIGURE 3:**
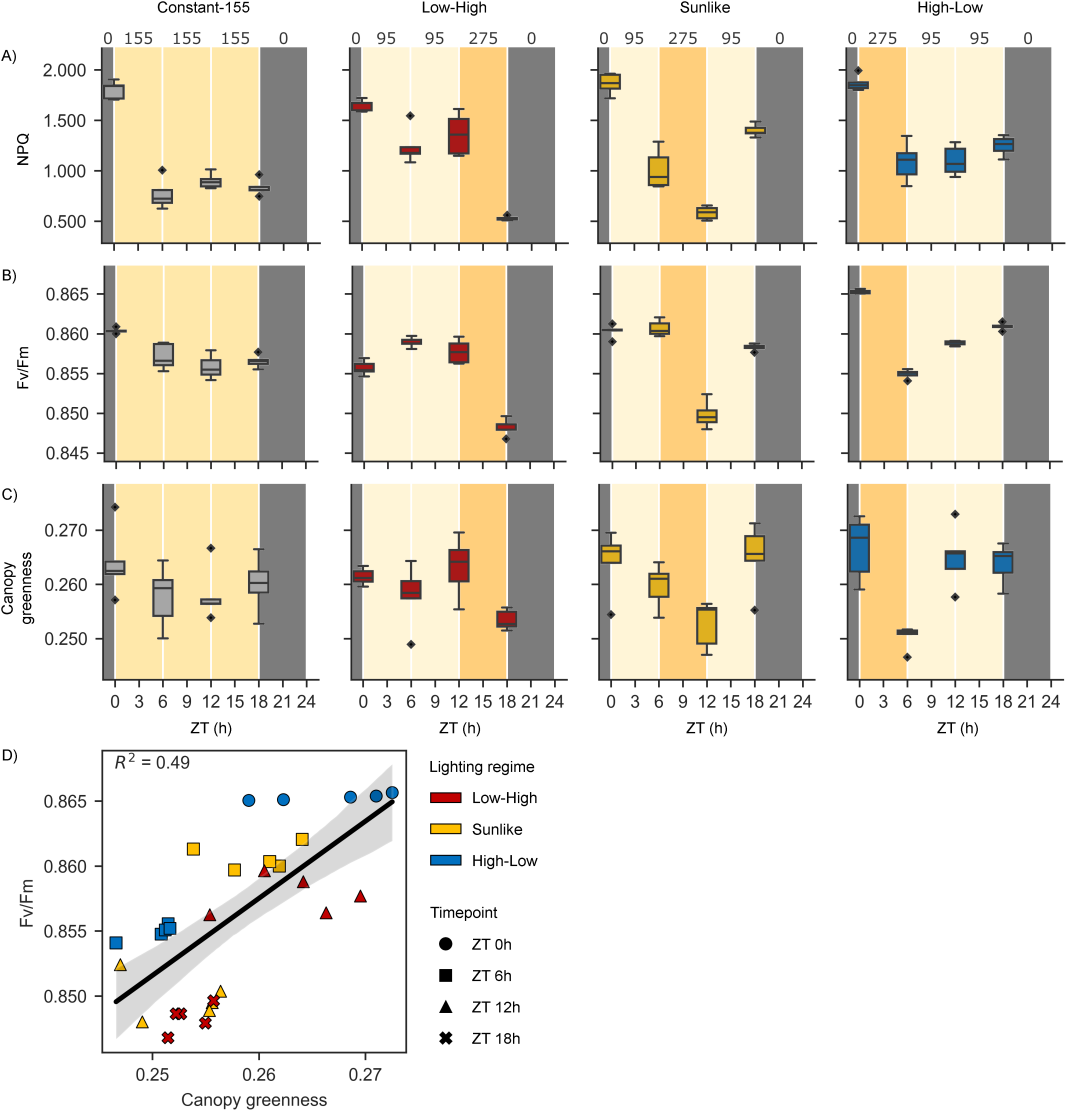
Dynamic regulation of photosynthetic traits in lettuce cv. “Katusa” grown under Constant‐155, Low‐High, Sunlike and High‐Low regimes in the plant phenotyping platform. Numbers on top of the panels and different shades of the background color indicate target light intensity values (PPFD). ZT (h) indicates time in hours elapsed since the beginning of the photoperiod. (A) NPQ, (B) Fv/Fm and (C) canopy greenness index was measured at four timepoints, 30 min before light intensity changes took place at 0, 6, 12 and 18 h ZT. (D) Correlation of Fv/Fm and canopy greenness between timepoints preceding and following a high light illumination period in lighting regimes Low‐High, Sunlike, and High‐Low. Symbol shapes and colors indicate measurement timepoints and lighting regimes, respectively. The photosynthetic parameters were analysed from a single experiment (*n* = 1) using five plants per lighting regime and timepoint. Pairwise comparison of photosynthetic parameters at a given timepoint in the different lighting regimes was conducted by ANOVA and Tukey HSD post hoc test with Bonferroni‐correction and is presented in Table 2.

The functional status of photosynthetic light reactions was first followed in the NaPPI phenotyping platform that enabled automated RGB and chlorophyll *a* fluorescence imaging under Constant‐155, Low‐High, Sunlike, and High‐Low lighting regimes. Under all dynamic lighting regimes, a small but consistent decrease was observed in NPQ, Fv/Fm, and canopy greenness index at the end of a 6‐h high light period, regardless of the time of day (Figure 3A–C, Table 2). Moreover, the analysis of the correlation between Fv/Fm and canopy greenness index upon transfer from low to high light intensity revealed that different lighting regimes formed distinct clusters (Figure 3D). This suggested that the dynamics of leaf greenness and photosynthetic efficiency were interconnected and sensitive to the lighting regime.

**TABLE 2:** Statistical analysis of data presented in Figure 3, depicting differences in chlorophyll fluorescence and canopy greenness when compared at each time point in plants grown in a plant phenotyping facility.

Timepoint	Lighting regime	NPQ		Fv/Fm		Canopy greenness	
ZT 0 h (05:45)	Constant‐155	1.802 ± 0.088	a	0.860 ± 0.0003	b	0.264 ± 0.006	a
	Low‐High	1.651 ± 0.056	b	0.856 ± 0.001	c	0.261 ± 0.002	a
	Sunlike	1.863 ± 0.101	a	0.860 ± 0.001	b	0.264 ± 0.006	a
	High‐Low	1.867 ± 0.076	a	0.865 ± 0.0002	a	0.267 ± 0.006	a
ZT 6 h (11:45)	Constant‐155	0.769 ± 0.148	b	0.857 ± 0.002	b	0.258 ± 0.006	ab
	Low‐High	1.242 ± 0.177	a	0.859 ± 0.001	ab	0.258 ± 0.006	ab
	Sunlike	1.013 ± 0.192	ab	0.861 ± 0.001	a	0.260 ± 0.004	a
	High‐Low	1.088 ± 0.192	ab	0.855 ± 0.001	c	0.250 ± 0.002	b
ZT 12 h (17:45)	Constant‐155	0.898 ± 0.073	b	0.856 ± 0.001	b	0.258 ± 0.005	ab
	Low‐High	1.361 ± 0.204	a	0.858 ± 0.001	ab	0.263 ± 0.005	a
	Sunlike	0.583 ± 0.064	c	0.850 ± 0.002	c	0.253 ± 0.004	b
	High‐Low	1.100 ± 0.147	b	0.859 ± 0.0003	a	0.265 ± 0.006	a
ZT 18 h (23:45)	Constant‐155	0.833 ± 0.079	c	0.857 ± 0.001	c	0.260 ± 0.005	ab
	Low‐High	0.527 ± 0.021	d	0.848 ± 0.001	d	0.253 ± 0.002	b
	Sunlike	1.398 ± 0.060	a	0.858 ± 0.0004	b	0.265 ± 0.006	a
	High‐Low	1.249 ± 0.096	b	0.861 ± 0.0004	a	0.264 ± 0.004	a
Lighting regime							
*p*‐value		< 0.0001		< 0.0001		0.44	
*F*‐value		13.66		82.07		0.91	
Timepoint							
*p*‐value		< 0.0001		< 0.0001		< 0.0001	
*F*‐value		203.36		102.94		8.33	
Lighting regime * timepoint							
*p*‐value		< 0.0001		< 0.0001		< 0.0001	
*F*‐value		29.49		90.14		5.35	

*Note:* Data was analyzed by ANOVA and Tukey HSD post hoc test with Bonferroni correction. Values indicate averages ± SD of five plants (*n* = 1). Values sharing the same letter within a column at a given time point are not significantly different. Treatment df = 3 and 40.

**FIGURE 4:**
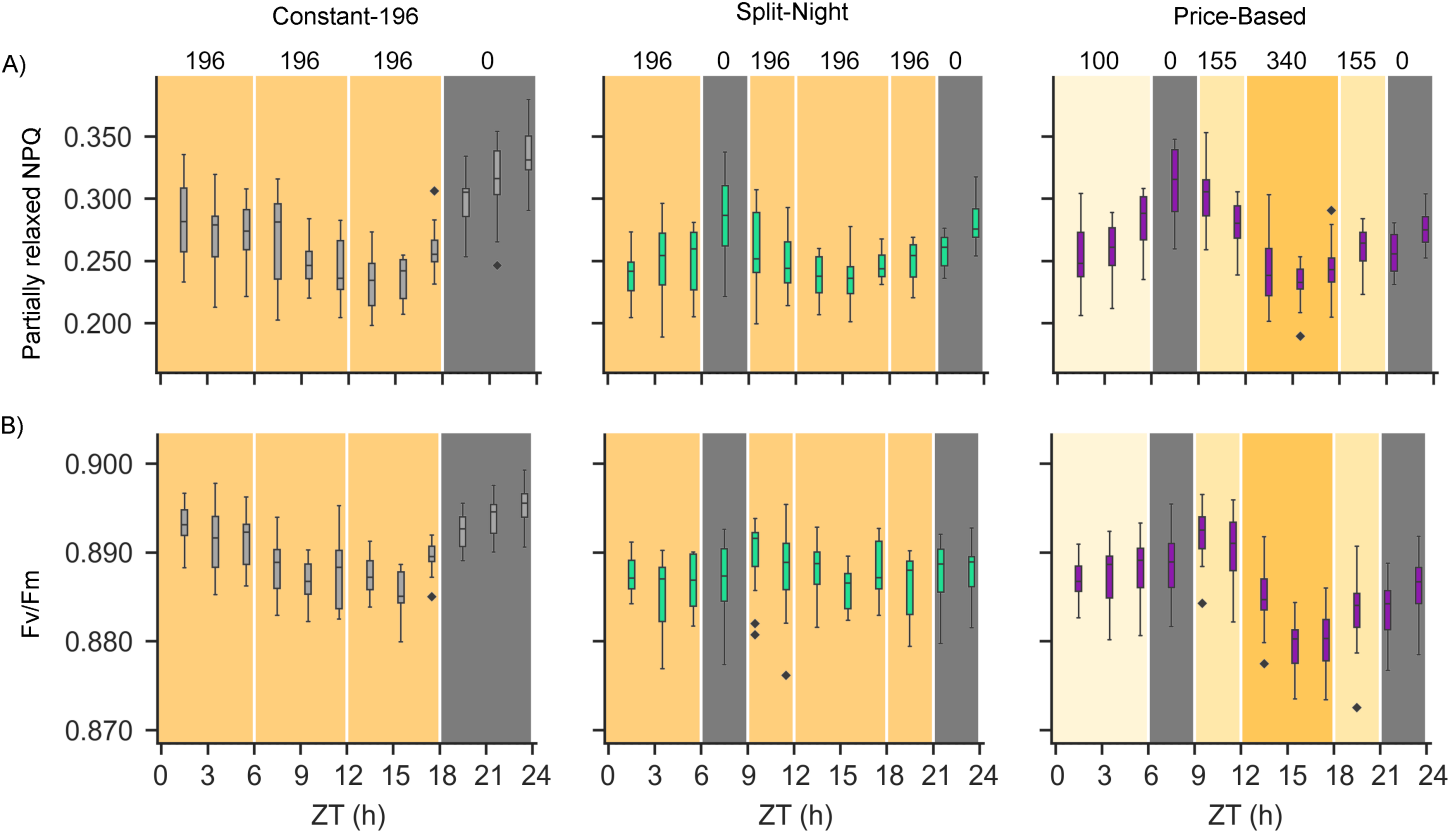
Dynamic regulation of photosynthetic traits in lettuce cv. “Katusa” measured at 2‐h intervals throughout the photoperiod in plants grown under Constant‐196, Split‐Night and Price‐Based lighting regimes in the larger‐scale vertical farming system. Numbers on top of the panels and different shades of the background color indicate target light intensity values (PPFD). ZT (h) indicates time in hours elapsed since the beginning of the photoperiod. (A) Partially relaxed NPQ and (B) Fv/Fm were imaged using a ChlF protocol including a 9‐min high light period at 800 μmol m^−2^ s^−1^ PPFD, followed by a 27‐min dark relaxation and then a light ramp consisting of 3‐min steps of increasing light intensity (100, 200, 300, 600, 800, 1000, and 1200 μmol m^−2^ s^−1^ PPFD). Saturating light pulses were triggered every 3 min throughout the protocol to determine Fv/Fm and NPQ. The photosynthetic parameters were analysed from three time‐independent experiments (*n* = 3) including 12 plants per lighting regime and timepoint. Pairwise comparison of photosynthetic parameters at a given timepoint in the different lighting regimes was conducted by a linear mixed effects model with random effects for grouping factors and is presented in Table 3.

For more detailed analysis of the effects of dynamic lighting on photosynthetic light reactions, chlorophyll *a* fluorescence imaging was performed every 2 h across the 24‐h period in the larger‐scale vertical farming system. This approach revealed similar patterns of fluctuation in Fv/Fm in the Constant‐155 and Constant‐196 lighting conditions (Figures 3 and 4). Another parameter following a similar pattern was the Partially relaxed NPQ that was calculated at 9 min into the dark relaxation period (Figures 3 and 4). Fluctuations in Fv/Fm and Partially relaxed NPQ were also observed under the bipartite Price‐Based lighting regime, but the pattern of fluctuation differed from that of the Constant‐196 plants (Figure 4, Table 3). Under Price‐Based conditions, Fv/Fm and Partially relaxed NPQ increased during the first 3‐h dark period but declined upon shift to light (Figure 4). In contrast, in the Split‐Night lighting regime, fluctuations in Fv/Fm and Partially relaxed NPQ were barely detectable (Figure 4). These results corroborated the finding that the photosynthetic light reactions can delicately adjust their function according to the lighting regime, without detrimental effects on lettuce growth and development (Figure 2H,I).

**TABLE 3:** Statistical analysis of data presented in Figure 4, depicting differences in chlorophyll fluorescence at each time point in plants grown in a larger‐scale vertical farm.

Timepoint	Lighting regime	Partially relaxed NPQ		Fv/Fm	
ZT 1.5 h (12:30)	Constant‐196	0.283 ± 0.032	a	0.893 ± 0.003	a
	Split‐Night	0.238 ± 0.016	c	0.888 ± 0.002	b
	Price‐Based	0.254 ± 0.024	b	0.887 ± 0.002	b
ZT 3.5 h (14:30)	Constant‐196	0.270 ± 0.029	a	0.891 ± 0.004	a
	Split‐Night	0.249 ± 0.030	b	0.886 ± 0.004	c
	Price‐Based	0.258 ± 0.022	b	0.887 ± 0.004	b
ZT 5.5 h (16:30)	Constant‐196	0.271 ± 0.024	a	0.891 ± 0.003	a
	Split‐Night	0.251 ± 0.026	c	0.887 ± 0.003	c
	Price‐Based	0.281 ± 0.024	b	0.888 ± 0.003	b
ZT 7.5 h (18:30)	Constant‐196	0.269 ± 0.035	a	0.888 ± 0.003	a
	Split‐Night	0.284 ± 0.035	c	0.887 ± 0.005	c
	Price‐Based	0.310 ± 0.030	b	0.889 ± 0.004	ab
ZT 9.5 h (20:30)	Constant‐196	0.248 ± 0.017	a	0.887 ± 0.002	a
	Split‐Night	0.260 ± 0.030	c	0.890 ± 0.004	c
	Price‐Based	0.303 ± 0.024	b	0.892 ± 0.003	b
ZT 11.5 h (22:30)	Constant‐196	0.244 ± 0.023	a	0.887 ± 0.004	a
	Split‐Night	0.247 ± 0.023	a	0.888 ± 0.005	a
	Price‐Based	0.277 ± 0.021	b	0.890 ± 0.004	b
ZT 13.5 h (00:30)	Constant‐196	0.233 ± 0.022	a	0.887 ± 0.003	a
	Split‐Night	0.237 ± 0.018	a	0.888 ± 0.003	a
	Price‐Based	0.242 ± 0.029	a	0.885 ± 0.003	b
ZT 15.5 h (02:30)	Constant‐196	0.236 ± 0.017	a	0.886 ± 0.003	a
	Split‐Night	0.237 ± 0.020	a	0.886 ± 0.002	a
	Price‐Based	0.232 ± 0.016	a	0.880 ± 0.003	b
ZT 17.5 h (04:30)	Constant‐196	0.259 ± 0.018	a	0.890 ± 0.002	a
	Split‐Night	0.246 ± 0.011	b	0.888 ± 0.003	a
	Price‐Based	0.245 ± 0.021	b	0.880 ± 0.004	b
ZT 19.5 h (06:30)	Constant‐196	0.296 ± 0.023	a	0.892 ± 0.002	a
	Split‐Night	0.250 ± 0.016	c	0.887 ± 0.004	c
	Price‐Based	0.260 ± 0.017	b	0.883 ± 0.004	b
ZT 21.5 h (08:30)	Constant‐196	0.315 ± 0.030	a	0.894 ± 0.002	a
	Split‐Night	0.258 ± 0.014	b	0.888 ± 0.003	c
	Price‐Based	0.256 ± 0.017	b	0.884 ± 0.003	b
ZT 23.5 h (10:30)	Constant‐196	0.333 ± 0.027	a	0.895 ± 0.002	a
	Split‐Night	0.280 ± 0.017	b	0.888 ± 0.003	c
	Price‐Based	0.275 ± 0.015	b	0.886 ± 0.004	b
Lighting regime					
*p*‐value		< 0.0001		< 0.0001	
*F*‐value		17		59.5	
Timepoint					
*p*‐value		0.0068		0.0183	
*F*‐value		7.39		5.6	
Lighting regime * timepoint					
*p*‐value		< 0.0001		< 0.0001	
*F*‐value		10.31		27.6	

*Note:* Data was analyzed using a linear mixed effects model with random effects for grouping factors. Values indicate averages ± SD of 12 plants (*n* = 3). Values sharing the same letter within a column at each timepoint are not significantly different. Treatment df = 2 and 415.

### 3.4 Accumulation and Depletion Patterns of Sucrose and Starch Follow the Cumulative Daily Light Integral, but the Overall Metabolic Composition of Lettuce cv. “Katusa” Leaves Remain Unaltered

**FIGURE 5:**
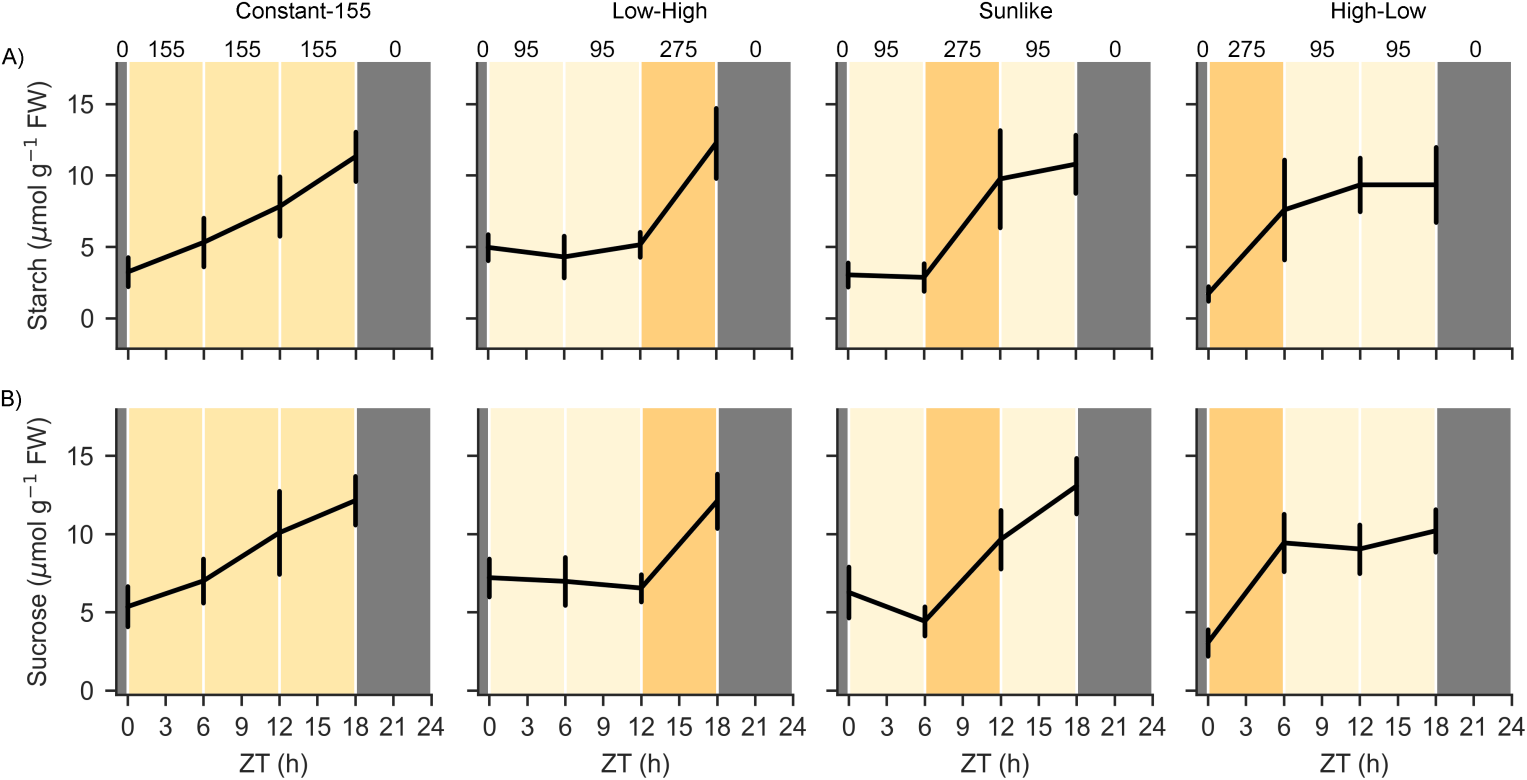
Contents of (A) starch and (B) sucrose (μmol g^−1^ FW) in lettuce cv. “Katusa” measured at timepoints ZT 0, 6, 12 and 18 h in plants grown under Constant‐155, Low‐High, Sunlike and High‐Low regimes in the small‐scale vertical farm testbed. Numbers on top of the panels and different shades of the background color indicate target light intensity values (PPFD). ZT (h) indicates time in hours elapsed since the beginning of the photoperiod. Data points indicate averages ± SD (*n* = 2). Starch and sucrose contents were analysed from two time‐independent experiments using 3–5 plants per lighting regime and timepoint. Pairwise comparison of sucrose, starch, fructose and glucose at a given timepoint in the different lighting regimes was conducted by a linear mixed effects model with random effects for grouping and is presented in Table 4.

**FIGURE 6:**
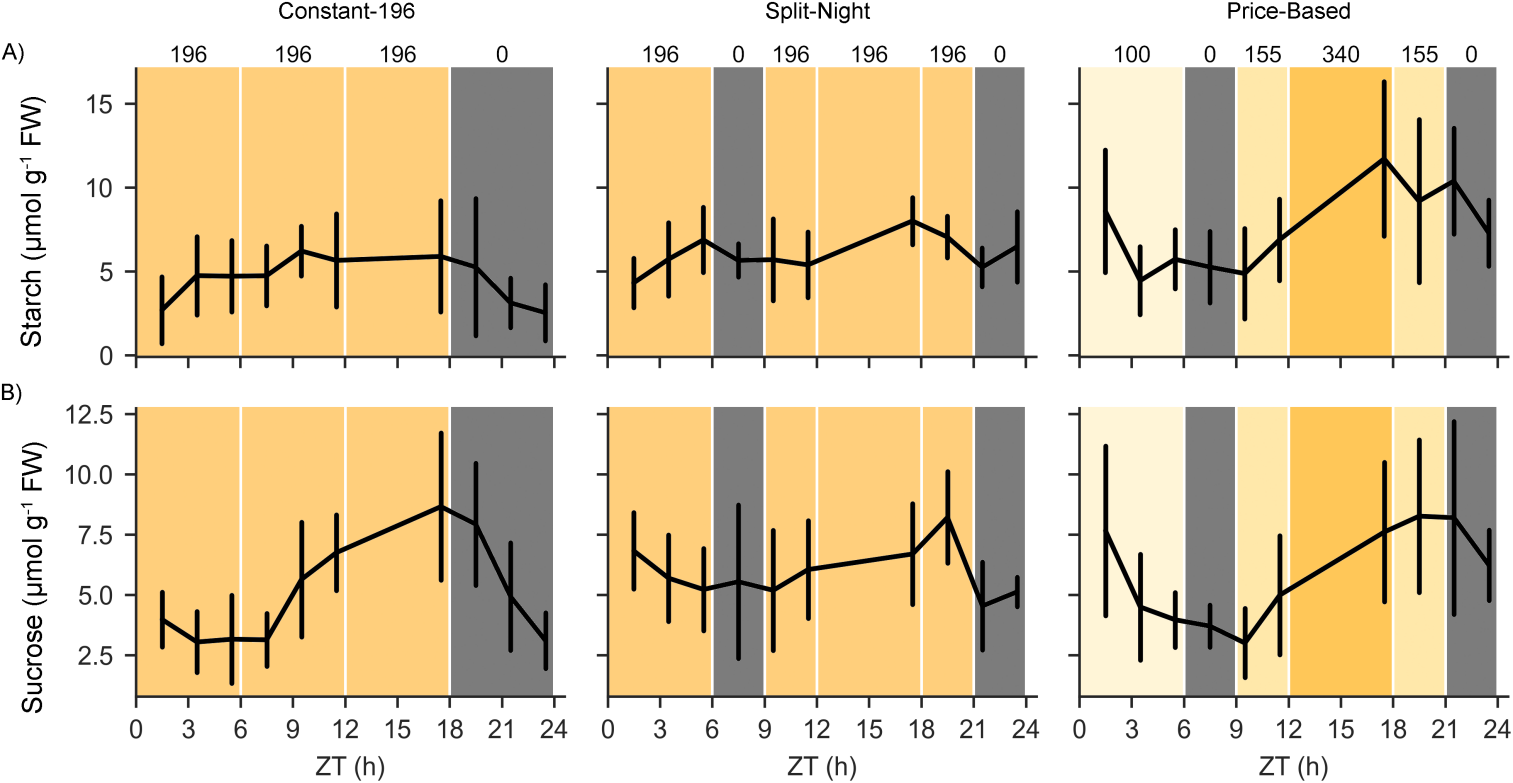
Contents of (A) starch and (B) sucrose (μmol g^−1^ FW) in lettuce cv. “Katusa” measured at 2‐h intervals throughout the photoperiod in plants grown under Constant‐196, Split‐Night and Price‐Based regimes in the larger‐scale vertical farming system. Numbers on top of the panels and different shades of the background color indicate target light intensity values (PPFD). ZT (h) indicates time in hours elapsed since the beginning of the photoperiod. Data points indicate averages ± SD (*n* = 3). Starch and sucrose contents were analysed from three time‐independent experiments including nine biological replicates per lighting regime and timepoint. Samples at timepoints 13.5 and 15.5 h ZT were omitted from the analysis due to measurement throughput capacity limitations. Pairwise comparison of starch, sucrose, fructose and glucose at a given timepoint in the different lighting regimes was conducted by a linear mixed effects model with random effects for grouping factors and is presented in Table 5.

To examine the photosynthetic production potential underlying growth under dynamic lighting, the accumulation and depletion patterns of sucrose and starch were analyzed across the 24‐h light/dark cycle. In plants grown under Constant‐155 in the small‐scale vertical farm testbed, the levels of sucrose and starch gradually increased until the end of the 18‐h photoperiod and decreased during the subsequent dark period (Figure 5, Table 4). Under the Low‐High regime, sucrose and starch contents increased slowly during the first 12 h of the photoperiod, and a clear increase was detected during the subsequent high‐light illumination period (Figure 5, Table 4). In Sunlike conditions, the levels of sucrose and starch increased during the high‐light period applied at midday (Figure 5, Table 4). In High‐Low, sucrose and starch contents increased rapidly during the early high‐light illumination period, but the accumulation slowed down over the rest of the photoperiod in low light intensity (Figure 5, Table 4). In all lighting regimes, the patterns of sucrose and starch contents were associated with the cumulative DLI, reaching similar concentration levels at the end of the photoperiod (Figures 1A and 5, Table 4). During the night, the levels of both sucrose and starch decreased. Parallel measurements of fructose and glucose contents did not reveal light quantity‐dependent patterns during the photoperiod (Table 4). Likewise, in the larger‐scale vertical farming experiment, the sucrose and starch contents followed changes occurring in the light intensity and differed between Constant‐196, Split‐Night, and Price‐Based regimes analyzed throughout the 24‐h photoperiod (Figure 6, Table 5).

**TABLE 4:** Statistical analysis depicting differences in photosynthetic end products when compared at different time points in lettuce cv.

Timepoint	Lighting regime	Starch (μmol g^−1^ FW)		Sucrose (μmol g^−1^ FW)		Fructose (μmol g^−1^ FW)		Glucose (μmol g^−1^ FW)	
ZT 0 h (05:45)	Constant‐155	3.23 ± 1.0	b	5.35 ± 1.2	b	6.53 ± 2.2	a	5.20 ± 2.1	a
	Low‐High	4.95 ± 0.9	a	7.21 ± 1.1	a	5.63 ± 1.4	ab	4.13 ± 1.3	a
	Sunlike	3.03 ± 0.8	b	6.27 ± 1.5	ab	4.70 ± 1.0	c	3.67 ± 1.1	b
	High‐Low	1.71 ± 0.5	c	3.04 ± 0.8	c	4.66 ± 1.1	bc	3.27 ± 1.0	b
ZT 6 h (11:45)	Constant‐155	5.31 ± 1.6	b	7.00 ± 1.3	b	6.76 ± 1.8	a	5.43 ± 1.4	a
	Low‐High	4.28 ± 1.4	bc	6.97 ± 1.4	b	6.83 ± 2.0	ab	5.56 ± 2.2	a
	Sunlike	2.85 ± 0.9	c	4.43 ± 0.9	c	5.34 ± 1.5	c	3.66 ± 1.0	b
	High‐Low	7.58 ± 3.3	a	9.43 ± 1.7	a	6.15 ± 1.7	bc	4.87 ± 1.8	b
ZT 12 h (17:45)	Constant‐155	7.82 ± 1.9	a	10.08 ± 2.5	a	5.58 ± 1.6	a	4.25 ± 0.9	a
	Low‐High	5.14 ± 0.8	b	6.54 ± 0.8	b	5.33 ± 1.4	ab	3.69 ± 1.5	a
	Sunlike	9.74 ± 3.2	a	9.65 ± 1.7	a	5.24 ± 1.9	c	3.67 ± 2.1	b
	High‐Low	9.33 ± 1.8	a	9.04 ± 1.5	a	5.07 ± 1.4	bc	3.14 ± 1.0	b
ZT 18 h (23:45)	Constant‐155	11.32 ± 1.6	a	12.14 ± 1.5	a	6.52 ± 0.6	a	5.34 ± 1.0	a
	Low‐High	12.25 ± 2.3	a	12.10 ± 1.6	a	6.41 ± 1.5	ab	5.29 ± 1.4	a
	Sunlike	10.78 ± 1.9	a	13.07 ± 1.7	a	5.50 ± 0.9	c	4.01 ± 1.2	b
	High‐Low	9.34 ± 2.5	a	10.20 ± 1.3	a	5.54 ± 1.1	bc	4.07 ± 1.0	b
Lighting regime									
*p*‐value		< 0.001		< 0.001		0.004		0.001	
*F*‐value		28.16		13.0		4.69		6.39	
Timepoint									
*p*‐value		< 0.0001		< 0.0001		0.011		0.003	
*F*‐value		126.3		111.1		3.90		5.04	
Lighting regime * timepoint									
*p*‐value		< 0.0001		< 0.0001		0.91		0.77	
*F*‐value		14.61		16.0		0.437		0.634	

*Note:* “Katusa” grown under Constant‐155, Low‐High, Sunlike and High‐Low lighting regimes in the small‐scale vertical farm testbed. Data was analysed using a linear mixed effects model with random effects for grouping factors. Values indicate averages ± SD of 3–5 plants (*n* = 2). Values sharing the same letter within a column at each timepoint are not significantly different. Starch treatment df = 3 and 104, sucrose, fructose and glucose treatment df = 3 and 105.

**TABLE 5:** Statistical analysis of data presented in Figure 6, depicting differences in photosynthetic end products at different time points in lettuce cv.

Timepoint	Lighting regime	Starch (μmol g^−1^ FW)		Sucrose (μmol g^−1^ FW)		Fructose (μmol g^−1^ FW)		Glucose (μmol g^−1^ FW)	
ZT 1.5 h (12:30)	Constant‐196	2.69 ± 2.00	a	3.97 ± 1.13	a	1.80 ± 0.95	a	1.11 ± 0.48	a
	Split‐Night	4.31 ± 1.47	c	6.82 ± 1.59	b	2.31 ± 0.65	a	1.30 ± 0.54	a
	Price‐Based	8.59 ± 3.66	b	7.65 ± 3.51	b	2.52 ± 1.15	a	1.26 ± 0.89	a
ZT 3.5 h (14:30)	Constant‐196	4.74 ± 2.35	a	3.04 ± 1.27	a	1.59 ± 0.64	ab	0.87 ± 0.72	a
	Split‐Night	5.72 ± 2.20	a	5.69 ± 1.80	c	2.22 ± 0.74	b	1.07 ± 0.55	a
	Price‐Based	4.44 ± 2.03	a	4.49 ± 2.19	b	1.09 ± 0.51	a	0.80 ± 0.74	a
ZT 5.5 h (16:30)	Constant‐196	4.71 ± 2.14	a	3.16 ± 1.83	a	1.95 ± 0.99	ab	0.99 ± 1.35	a
	Split‐Night	6.88 ± 1.95	a	5.22 ± 1.71	b	1.19 ± 0.47	b	0.70 ± 0.54	a
	Price‐Based	5.72 ± 1.76	a	3.96 ± 1.14	ab	2.34 ± 1.45	a	1.35 ± 0.82	a
ZT 7.5 h (18:30)	Constant‐196	4.74 ± 1.79	a	3.13 ± 1.10	a	1.86 ± 1.35	a	1.12 ± 0.57	a
	Split‐Night	5.65 ± 1.00	a	5.54 ± 3.19	b	2.47 ± 2.27	a	1.43 ± 0.78	a
	Price‐Based	5.26 ± 2.14	a	3.70 ± 0.88	b	1.97 ± 0.93	a	1.19 ± 1.09	a
ZT 9.5 h (20:30)	Constant‐196	6.21 ± 1.48	a	5.64 ± 2.38	a	2.11 ± 1.15	a	1.48 ± 0.53	a
	Split‐Night	5.69 ± 2.43	a	5.18 ± 2.49	ab	1.46 ± 1.02	a	0.67 ± 0.47	a
	Price‐Based	4.87 ± 2.70	a	3.00 ± 1.43	b	1.56 ± 1.52	b	1.05 ± 0.86	a
ZT 11.5 h (22:30)	Constant‐196	5.65 ± 2.78	a	6.75 ± 1.57	a	1.85 ± 1.51	a	1.15 ± 0.74	a
	Split‐Night	5.39 ± 1.96	a	6.04 ± 2.03	a	1.45 ± 1.11	a	0.89 ± 0.74	a
	Price‐Based	6.89 ± 2.43	a	4.98 ± 2.46	a	1.75 ± 1.49	a	1.22 ± 0.82	a
ZT 13.5 h (00:30)	Constant‐196	n.a.		n.a.		n.a.		n.a.	
	Split‐Night	n.a.		n.a.		n.a.		n.a.	
	Price‐Based	n.a.		n.a.		n.a.		n.a.	
ZT 15.5 h (02:30)	Constant‐196	n.a.		n.a.		n.a.		n.a.	
	Split‐Night	n.a.		n.a.		n.a.		n.a.	
	Price‐Based	n.a.		n.a.		n.a.		n.a.	
ZT 17.5 h (04:30)	Constant‐196	5.89 ± 3.32	a	8.65 ± 3.05	a	1.61 ± 0.74	a	0.74 ± 0.63	a
	Split‐Night	8.00 ± 1.40	c	6.69 ± 2.10	c	2.03 ± 1.25	ab	0.58 ± 0.30	a
	Price‐Based	11.72 ± 4.62	b	7.60 ± 2.89	b	2.50 ± 1.16	b	1.33 ± 0.52	a
ZT 19.5 h (06:30)	Constant‐196	5.26 ± 4.11	a	7.92 ± 2.53	a	1.45 ± 1.26	a	0.49 ± 0.37	a
	Split‐Night	7.05 ± 1.24	b	8.20 ± 1.90	a	2.03 ± 1.25	a	1.09 ± 0.63	a
	Price‐Based	9.19 ± 4.86	b	8.26 ± 3.16	a	2.20 ± 1.03	a	0.80 ± 0.65	a
ZT 21.5 h (08:30)	Constant‐196	3.12 ± 1.49	a	4.92 ± 2.24	a	2.03 ± 1.25	a	0.96 ± 0.65	a
	Split‐Night	5.24 ± 1.17	c	4.53 ± 1.82	a	1.94 ± 1.24	a	0.82 ± 0.61	a
	Price‐Based	10.39 ± 3.17	b	8.19 ± 4.00	b	2.75 ± 2.17	a	1.48 ± 1.46	a
ZT 23.5 h (10:30)	Constant‐196	2.53 ± 1.68	a	3.10 ± 1.67	a	1.92 ± 1.67	a	0.91 ± 0.80	a
	Split‐Night	6.47 ± 2.11	b	5.12 ± 0.61	c	1.81 ± 1.03	a	0.82 ± 0.33	a
	Price‐Based	7.29 ± 1.98	b	6.23 ± 1.46	b	2.36 ± 1.24	a	0.68 ± 0.35	a
Lighting regime									
*p*‐value		< 0.0001		0.046		0.177		0.094	
*F*‐value		25.87		3.12		1.74		2.39	
Timepoint									
*p*‐value		0.0021		< 0.0001		0.043		0.030	
*F*‐value		9.66		39.04		4.15		4.76	
Lighting regime * timepoint									
*p*‐value		< 0.0001		0.003		0.011		0.12	
*F*‐value		14.15		5.84		4.63		2.18	

*Note:* “Katusa” grown under Constant‐196, Split‐Night and Price‐Based regimes in the larger‐scale vertical farm. Data was analysed using a linear mixed effects model with random effects for grouping factors. Values indicate averages ± SD of nine plants (*n* = 3). Values sharing the same letter within a column at each timepoint are not significantly different. Starch treatment df = 2 and 254, sucrose and fructose treatment df = 2 and 248, glucose treatment df = 2 and 243. Samples at timepoints 13.5 h and 15.5 h ZT were omitted from the analysis due to measurement throughput capacity limitations.

**FIGURE 7:**
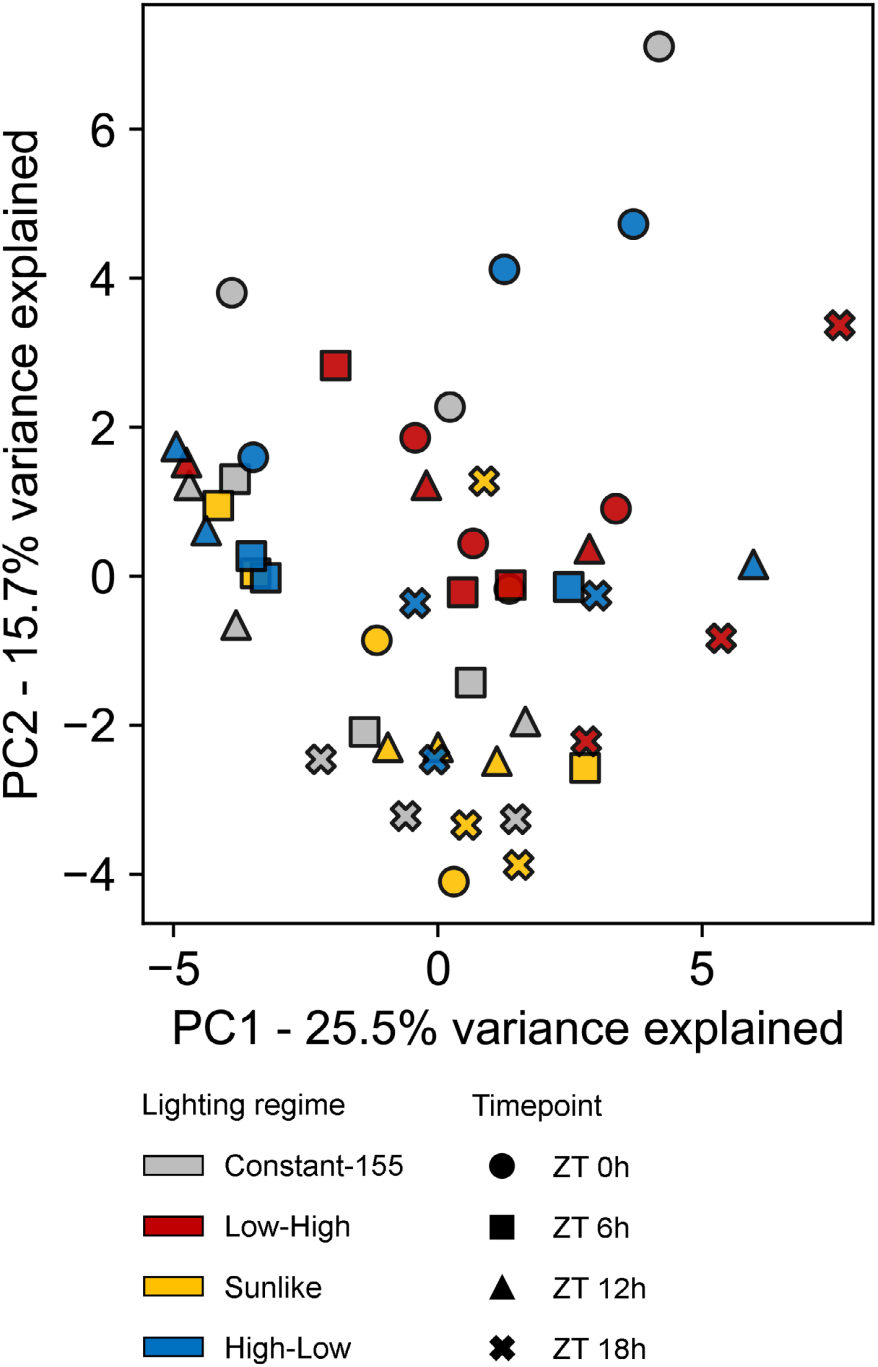
Principal component analysis (PCA) of metabolite profiles in lettuce cv. “Katusa” grown under Constant‐155, Low‐High, Sunlike and High‐Low regimes in the small‐scale vertical farm testbed. The samples were collected 15 min before light intensity changes took place at timepoints ZT 0, 6, 12 and 18 h. Each symbol represents a biological replicate, consisting of a pool of three plants. Symbol colors and shapes indicate lighting regimes and measurement timepoints, respectively.

Finally, untargeted metabolite profiling across the 24‐h light/dark cycle was conducted to examine whether growth under dynamic lighting regimes induced wider metabolic shifts in plants grown in the small‐scale vertical farming testbed. Altogether, 199 compounds were detected using negative mode and 271 compounds using positive mode in MS analysis (Data S2). The identified compounds included aromatic amino acids, panthotenic acid (vitamin B5), various phenolic compounds including caffeic acid, quercetin, kaempherol derivatives, and sesquiterpenoid lactones (Data S2). Multivariate analysis of metabolite profiles did not reveal clustering among sample sets (Figure 7). Rather, the slight variations between individual samples suggested that growth under moderately alternating dynamic lighting did not deteriorate the metabolite content of lettuce cv. “Katusa” leaves (Figure 7).

## 4 Discussion

Dynamic LED‐based lighting technologies could forward CEA by enabling cost‐effective growth without compromising the yield or nutritional quality of the produce. Our estimation of electricity costs using average spot‐price information from NordPool for 2020–2023 indicated up to 38.3% cost savings under dynamic lighting (Figure 1C, Table 1). These savings are in line with studies that modeled and experimentally validated potential electricity cost savings of shifting LED light use toward lower demand hours (Avgoustaki and Xydis 2021; Arabzadeh et al. 2023). Achieving profitable crop production under dynamic lighting requires crop species‐specific knowledge on plant growth and development under different lighting conditions, as well as understanding the balance between power grid supply, demand and distribution (Arabzadeh et al. 2023). We found that lettuce cv. “Katusa” readily adjusted its photosynthetic processes according to the prevailing light intensity throughout the photoperiod (Figures 3, 4, 5, 6), enabling growth and productivity under dynamic lighting regimes (Figure 2). Lettuce is among the most cultivated leafy vegetables in CEA and comprises a feasible model plant for physiological studies. Future research should integrate data from different crop species and genetically distinct cultivars to uncover the molecular mechanisms underlying tolerance to dynamic lighting among horticultural crops.

### 4.1 Dynamic Lighting Conditions Maintain Lettuce Growth in Different Indoor Cultivation Setups

In our experiments, the shifting light intensity periods in the three different setups were programmed to occur repeatedly at specific times of the photoperiod, and the magnitude of differences between low and high light intensities was kept at a maximum of 3.4‐fold to avoid light‐induced stress (Figures 1, 3 and 4). In these conditions, lettuce cv. “Katusa” grew comparatively well both under constant and dynamic lighting regimes, indicating that DLI can be redistributed across the photoperiod in different ways (Figure 2). These findings were in line with Poorter et al. (2019), who conducted a larger‐scale meta‐analysis of 500 experiments encompassing 760 plant species and found that physiological, developmental, and chemical plant traits were all responsive to the DLI.

Understanding how changing light conditions affect growth in different species is key to optimizing growth conditions for commercial production. Earlier studies have suggested that drastic changes in light conditions could impede plant growth (Morales and Kaiser 2020; Bhuiyan and van Iersel 2021). Studies on the model species *A. thaliana* , aimed at identification of photoprotective components, were often designed to cause light stress with periods of low and high light alternating on a time scale of minutes (Suorsa et al. 2012; Kono et al. 2017; Garcia‐Molina and Leister 2020). The outcomes of laboratory studies suggested that dynamic acclimation to changing light intensities involves regulatory interactions among photosynthetic reactions, photoprotective mechanisms, leaf development, and diurnal physiological processes (Schneider et al. 2019; Niu et al. 2024; Shikanai 2024), which should match the changing light intensities to maintain growth. Recently, Kaiser et al. (2024) tested different leafy vegetables and herbs, including basil ( *Ocimum basilicum* ), pak choi ( *Brassica rapa* subsp. *chinensis*), rucola ( *Diplotaxis tenuifolia* ), and spinach ( *Spinacia oleracea* ) under hourly alterations in light intensities, and showed that their marketable FW were not affected when compared to plants grown under constant light conditions of equal DLI.

We took advantage of a phenotyping platform equipped with adjustable LED luminaires and imaging systems to validate lettuce growth under dynamic lighting. Lettuce displayed similar developmental responses with no undesirable morphological alterations under dynamic vs. constant lighting regimes, even though light intensities changed at different times of the day (Figure 2). Likewise, Bochenek and Fallstrom (2016) and Bhuiyan and van Iersel (2021) found that moderate light intensity changes causing temporal differences in distribution of the DLI did not cause growth defects in biomass accumulation of lettuce cv. “Galiano”, “Little Gem” or “Green Salad Bowl.” In a larger‐scale vertical farm, simulating commercial production with a hydroponic NFT growth system and supplemental CO_2_, leaf biomass accumulation under dynamic lighting did not differ from constant light controls (Figures 1B and 2H). This was observed even though the photoperiods were interrupted by two periods of darkness under Split‐Night and Price‐Based lighting regimes (Figures 1B, 2H,I). These conditions, designed to simulate energy‐efficient dynamic lighting in commercial production, supported lettuce cv. ‘Katusa’ growth (Figure 2H,I) but did not induce flowering, suggesting scalability of the dynamic lighting strategies.

In our experiments, shifts between light and dark as well as transitions between light intensity levels during the photoperiod were instantaneous and abrupt (Figure 1A,B). In natural lighting conditions, however, diurnal changes in light intensity occur gradually over time on timescales varying from minutes to hours. Momentary changes in electricity prices, in turn, may sometimes be highly stochastic and follow an entirely different rhythm, primarily defined by the principles of the local electricity grid (supply) capacity and consumption (demand). On a general level, however, the prices typically follow a repeating pattern characterized by distinct higher and lower demand hours, with gradual transitions in‐between (Figure 1C). For dynamic control of LED lights in indoor farming, this presents challenges and opportunities for fine‐tuning the lighting regime profiles to account for the needs of plants. Twilight length, or the duration of light intensity transitions at dawn and dusk, has been found to influence plant growth and flowering time in Arabidopsis via the *LHY/CCA1*‐circadian clock pathway (Mehta et al. 2024). Plants grown under photoperiods with twilight lengths between 30 and 60‐min grew larger than plants in no‐twilight photoperiods (Mehta et al. 2024). As precise control of light intensity over time can be achieved with LED luminaires, further studies should examine how gradual transitions in light intensity influence plant development and growth in CEA. Additionally, sophisticated crop monitoring and modeling should be used to optimize plant growth and energy use efficiency in vertical farming systems.

### 4.2 Photosynthetic Metabolism Responds to Dynamically Changing Lighting, While the Overall Leaf Chemical Composition Does Not Show Short‐Term Fluctuations in Response to Light

We utilized chlorophyll fluorescence imaging in combination with metabolite profiling to address the physiological and biochemical responses underlying lettuce cv. “Katusa” growth under dynamic lighting. By comparing photosynthetic responses in a timescale of hours, we found that photosynthetic light reactions and carbon metabolism were delicately balanced according to the duration and intensity of dynamic lighting in lettuce cv. “Katusa” (Figures 3, 4, 5, 6, Tables 2, 3, 4, 5).

In our dynamic lighting conditions, NPQ decreased during the high light illumination period in Low‐High, Sunlike, and High‐Low regimes, suggesting photochemical quenching of chlorophyll fluorescence via carbon metabolism, which could drain reducing equivalents from the photosynthetic electron transfer chain and promote accumulation of sucrose and starch (Figure 3A, Table 2). Photobiological studies have suggested that plants undergo different photosynthetic adjustments to fluctuating light conditions, depending on the frequency, duration, and intensity of fluctuating light (Von Bismarck et al. 2023; Vialet‐Chabrand et al. 2017; Alter et al. 2012; Yin and Johnson 2000; Lazzarin et al. 2024). Our dynamic lighting conditions comprised one daily high light period with up to 3.4‐fold differences in light intensities (Figure 1A,B). The dynamic lighting conditions did not result in decreased photosynthetic productivity when compared to constant light with equal DLI (Figures 1, 2, 3, 4, 5, 6), suggesting that metabolic interactions were sufficient to allow dynamic acclimation according to the prevailing lighting.

Following the functional status of photosynthetic light reactions with higher temporal resolution in the larger‐scale vertical farming system revealed delicate fluctuations in photosynthetic parameters, which could be detected by chlorophyll fluorescence imaging under the more complex, alternative day/night regimes, such as the Split‐Night and Price‐Based lighting regimes (Figures 1B and 4, Table 3). The ability for dynamic photosynthetic adjustments is important, since delays in light‐induced adjustments of photosynthetic gas exchange, carbon assimilation, or balancing of the photosynthetic light reactions could negatively affect photosynthetic activity and yield (Kromdijk et al. 2016; Taylor and Long 2017). A well‐known example critical under natural conditions is slow relaxation of NPQ upon rapid shifts from high to low light intensity, which prolongs the dissipation of excitation energy, thereby limiting the conversion of light into chemical energy needed for photosynthesis (Kromdijk et al. 2016; De Souza et al. 2022; Niu et al. 2023). Excessive dissipation of excitation energy via NPQ could equally well limit the availability of light energy for photochemistry, thereby limiting resource allocation for growth and development. Our findings suggest that the photosynthetic regulatory circuits of lettuce can cope with light intensity changes introduced on a timescale of hours, without detrimental effects on the accumulation of leaf biomass (Figures 2 and 4). Artificial day/night regimes could therefore offer tools to modulate photosynthetic limitations to plant production. The impact of NPQ relaxation rates on plant biomass accumulation was demonstrated under fluctuating light conditions with minute‐scale intensity changes (Kromdijk et al. 2016; De Souza et al. 2022). Future research should explore how modifying NPQ relaxation rates through lighting regimes like Split‐Night or Price‐Based (Figure 4) influences plant growth under rapidly changing light conditions.

Comparison of metabolite profiles between plants grown under constant moderate light vs. high light has revealed high‐light‐induced metabolic responses in model plants and horticultural crop species (Jänkänpää et al. 2012; Ishihara et al. 2025). Changes in primary metabolism as well as accumulation of phenolic compounds, notably anthocyanins and flavonoids, are important responses that can protect plants against potentially damaging effects of light. It is noteworthy that the metabolic responses observed upon growth of plants under constant moderate or high light may stem from the differential DLI received by the plants (Ishihara et al. 2025). In our experiments, lettuce cv. ‘Katusa’ grown under varying light regimes, but equal DLI, displayed photosynthetic adjustments that followed the cumulative DLI, whereas overall metabolite profiles did not display dynamic light‐induced alterations during the photoperiod (Figure 7).

In conclusion, the results of this study suggested that the application of dynamic lighting regimes can support lettuce growth without impairing leaf chemical composition. Understanding the dynamics of plant metabolism and overcoming photosynthetic limitations to plant production under changing light intensities are keys to cost‐effective indoor cultivation.

## Author Contributions

Conceptualization: Arttu Mäkinen, Alexey Shapiguzov, Titta Kotilainen, Saijaliisa Kangasjärvi, Paula Elomaa, Sylvain Poque. Methodologies: Arttu Mäkinen, Alexey Shapiguzov, Sylvain Poque, Hirofumi Ishihara, Nina Sipari. Software/VF design and setup: Arttu Mäkinen, Titta Kotilainen; Matti Pastell. Investigation: Arttu Mäkinen, Sylvain Poque, Hirofumi Ishihara, Alexey Shapiguzov, Titta Kotilainen, Ilona Varjus. Validation: Arttu Mäkinen, Sylvain Poque, Alexey Shapiguzov, Nina Sipari, Juho Heininen. Formal analysis: Nina Sipari, Juho Heininen, Sylvain Poque. Data curation: Arttu Mäkinen, Nina Sipari, Juho Heininen, Sylvain Poque, Titta Kotilainen. Visualization: Sylvain Poque; Writing – original draft, Arttu Mäkinen, Alexey Shapiguzov, Sylvain Poque, Nina Sipari, Titta Kotilainen, Saijaliisa Kangasjärvi. Writing – review and editing: Arttu Mäkinen, Saijaliisa Kangasjärvi, Titta Kotilainen, Paula Elomaa, Kristiina Himanen. Supervision: Saijaliisa Kangasjärvi, Paula Elomaa. Project administration: Saijaliisa Kangasjärvi. Resources: Saijaliisa Kangasjärvi, Kristiina Himanen, Matti Pastell. Funding acquisition: Saijaliisa Kangasjärvi, Paula Elomaa, Arttu Mäkinen, Matti Pastell.

## Conflicts of Interest

The authors declare no conflicts of interest.

## Supporting information


**Figure S1.** Spectral distributions and photon flux density per wavelength area in different indoor cultivation setups.
**Table S1.** Growing conditions and indoor cultivation setups used in this study.

**Data S1.** Raw data of all figures and tables.

**Data S2.** Large‐scale metabolite profiling of lettuce cv. “Katusa” grown under dynamic.

## Data Availability

Data that supports the findings of this study are available in the Supporting Information of this article.
