# Supplementary material for: Photosynthetic Adjustments Maintain Lettuce Growth Under Dynamically Changing Lighting in Controlled Indoor Farming Setups
Source: Physiol Plant. 2025 Jul 12;177(4):e70405. doi: 10.1111/ppl.70405 (PMC12254939; doi:10.1111/ppl.70405)
Supplement: Supplementary file 1 — Figure S1. Spectral distributions and photon flux density per wavelength area in different indoor cultivation setups. Table S1. Growing conditions and indoor cultivation setups used in this study. [file PPL-177-e70405-s002.pdf]

Photosynthetic adjustments maintain lettuce growth under dynamically changing lighting in controlled indoor farming setups

Arttu Mäkinen

Supplemental Table S1. Growing conditions and indoor cultivation setups.

Part 1: Seedling phase growing conditions.

Parameter	Small-scale vertical farming experiment	Plant phenotyping experiment	Large-scale vertical farming experiment
Seedling phase duration	9 days	12 days	Repetition 1: 13 days<br>Repetition 2: 12 days<br>Repetition 3: 14 days
Plant material	*Lactuca sativa* L., cv. 'Katusa'	*Lactuca sativa* L., cv. 'Katusa'	*Lactuca sativa* L., cv. 'Katusa'
Growth medium	1:1-mix of peat (White 420 W B2, Kekkilä-BVB Oy, Vantaa, Finland) and vermiculite (RHP Agra-Vermiculite, grain size F2/0-3 mm, RHP, 's-Gravenzande, the Netherlands)	1:1 mix of peat (W HS R8017, Kekkilä-BVB Oy, Vantaa, Finland) and vermiculite (RHP Agra-Vermiculite, grain size F2/0-3 mm, RHP, 's-Gravenzande, the Netherlands)	fine peat (Kekkilä Professional, VHM 620 R8060, Kekkilä Oy, Vantaa, Finland)
Pot size	385 ml<br>(Ø 8 cm, one seed per pot)	250 ml<br>(Ø 6 cm, one seed per pot)	80 ml<br>(Ø 5 cm, one seed per pot)
Pot quantity	Repetition 1: 48 pcs<br>Repetition 2: 96 pcs<br>Repetition 3: 80 pcs	80 pcs	Repetition 1: 264 pcs<br>Repetition 2: 264 pcs<br>Repetition 3: 264 pcs
Fertilizer	1 g/ L (Kekkilä Garden Kastelulannoite, NPK: 17-4-25, Kekkilä-BVB Oy, Vantaa, Finland)	1 ml / L (Neko Kasviravinne, NPK: 7-2-2, Oy Neko Ab, Hämeenlinna, Finland)	EC 1.8 dS m^{-1}, pH 6 mixture of Vihannes Superex (NPK: 9-5-31, Kekkilä Professional, Kekkilä Oy, Finland) and YaraTera Calcinit (N: 15.5, Yara Suomi Oy, Finland)
Stratification conditions	72h in darkness at +6°C	72h in darkness at +6°C	24h in darkness at +18°C
Air temperature	Day: 21°C<br>Night: 19°C	Day: 21°C<br>Night: 19°C	Day: 18°C<br>Night: 18°C
Relative humidity	40-60%	60-70%	60-80%
CO_2-concentration	ambient	ambient	800 ppm
Location	walk-in growth room	walk-in growth room (FytoScope™, Photon Systems Instruments, PSI, Drásov, Czech Republic)	greenhouse
Irrigation	manual irrigation	automatic irrigation	manual irrigation
Photoperiod	L18:D6	L18:D6	L18:D6
Light intensity levels	155 µmol m^{-2} s	155 µmol m^{-2} s	350 µmol m^{-2} s
Daily light integral	10 mol m^{-2}	10 mol m^{-2}	22.7 mol m^{-2}
Light spectrum	Valoya BX120 AP673L (Valoya Greenlux Lighting Solutions Oy, Helsinki, Finland)	PSI CS 250/300_4_2.4 (Photon Systems Instruments, PSI, Drásov, Czech Republic)	VYPR PhysioSpec Greenhouse (Fluence Bioengineering Inc., Austin TX, USA)

Part 2: Experiment phase growing conditions.

Parameter	Small-scale vertical farming experiment				Plant phenotyping experiment				Large-scale vertical farming experiment		
Experiment phase duration	14 days				17 days				Repetition 1: 14 days<br>Repetition 2: 21 days<br>Repetition 3: 21 days		
Lighting regimes	Constant-155	Low-High	Sunlike	High-Low	Constant-155	Low-High	Sunlike	High-Low	Constant-196	Split-Night	Price-Based
Light intensity levels (µmol m^{-2} s)	155	95, 275	95, 275	95, 275	155	95, 275	95, 275	95, 275	196	196	100, 155, 340
Photoperiod	L18:D6				L18:D6				L18: D6	L6: D3: L12: D3	L6: D3: L12: D3
Daily light integral	10 mol m^{-2}				10 mol m^{-2}				12.7 mol m^{-2}		
Light spectrum	Valoya BX120 AP673L (Valoya Greenlux Lighting Solutions Oy, Helsinki, Finland)				PSI CS 250/300_4_2.4 (Photon Systems Instruments, Drásov, Czech Republic)				Valoya BX120 Solray (Valoya Greenlux Lighting Solutions Oy, Helsinki, Finland)		
Location	walk-in growth room				walk-in growth room (FytoScope^{TM}, Photon Systems Instruments, Drásov, Czech Republic)				walk-in growth room (VIS-experimental growth room, Vacuum Insulation Solutions Oy, Espoo, Finland)		
Plant density (plants / m^{-2})	Repetition 1: 15.4<br>Repetition 2: 30.8<br>Repetition 3: 25.6				45.7				10		
Irrigation	manual tray irrigation				automatic irrigation				nutrient film-technique (NFT)		
Growth medium	same as in seedling phase				same as in seedling phase				same as in seedling phase		
Pot size	same as in seedling phase				same as in seedling phase				same as in seedling phase		
Pot quantity	same as in seedling phase				same as in seedling phase				same as in seedling phase		
Fertilizer	same as in seedling phase				no fertilizer				same as in seedling phase		
Air temperature	same as in seedling phase				same as in seedling phase				same as in seedling phase		
Relative humidity	same as in seedling phase				same as in seedling phase				same as in seedling phase		
CO_2-concentration	same as in seedling phase				same as in seedling phase				same as in seedling phase		

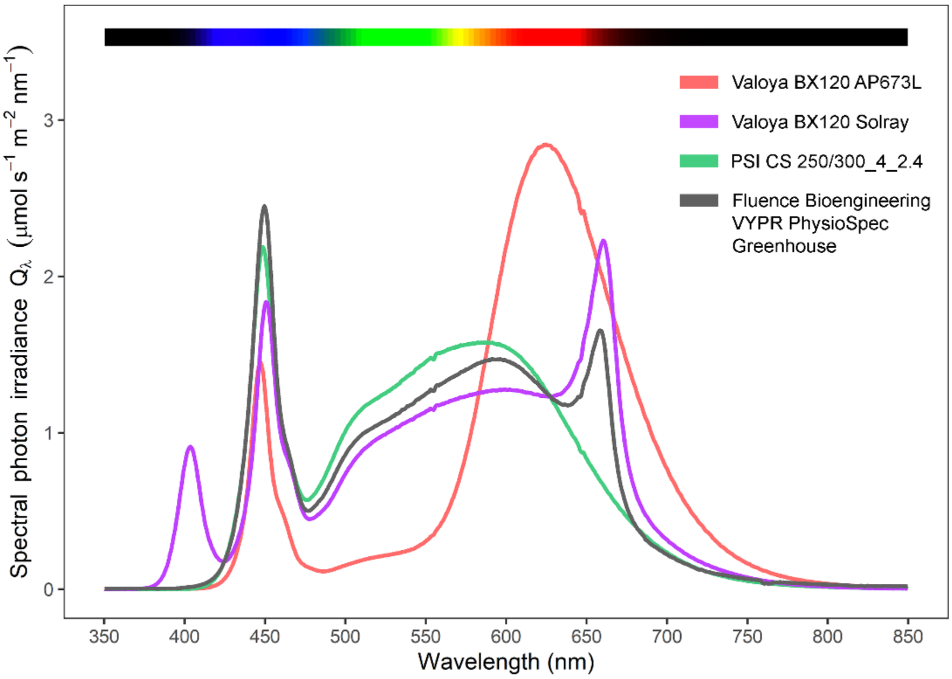

Supplemental Figure 1. Spectral distributions and photon flux density per wavelength area in different indoor cultivation setups.
